# Empirical Bayes method for reducing false discovery rates of correlation matrices with block diagonal structure

**DOI:** 10.1186/s12859-017-1623-y

**Published:** 2017-04-12

**Authors:** Clare Pacini, James W. Ajioka, Gos Micklem

**Affiliations:** 1 CCBI, Department Applied Mathematics and Theoretical Physics, University of Cambridge, Wilberforce Road, Cambridge, CB3 0WA UK; 2 Department of Pathology, University of Cambridge, Tennis Court Road, Cambridge, CB2 1QP UK; 3 Cambridge Systems Biology Centre, University of Cambridge, Tennis Court Road, Cambridge, CB2 1QR UK

**Keywords:** Empirical Bayes, Correlation

## Abstract

**Background:**

Correlation matrices are important in inferring relationships and networks between regulatory or signalling elements in biological systems. With currently available technology sample sizes for experiments are typically small, meaning that these correlations can be difficult to estimate. At a genome-wide scale estimation of correlation matrices can also be computationally demanding.

**Results:**

We develop an empirical Bayes approach to improve covariance estimates for gene expression, where we assume the covariance matrix takes a block diagonal form. Our method shows lower false discovery rates than existing methods on simulated data. Applied to a real data set from *Bacillus subtilis* we demonstrate it’s ability to detecting known regulatory units and interactions between them.

**Conclusions:**

We demonstrate that, compared to existing methods, our method is able to find significant covariances and also to control false discovery rates, even when the sample size is small (*n*=10). The method can be used to find potential regulatory networks, and it may also be used as a pre-processing step for methods that calculate, for example, partial correlations, so enabling the inference of the causal and hierarchical structure of the networks.

## Background

Correlation analysis is a common approach for identifying relationships between the expression of genes or proteins. Initially the covariance matrix would be calculated which may then be either inverted to find a partial correlation matrix, or standardised to give the correlation matrix. The correlation matrix is often used in downstream analysis such as graphical inference or using partial correlations, that further provides causal information. One problem with this type of analysis is that often, by necessity, sample sizes are small and this reduces the power of detecting correlations and increases the false discovery rate.

Methods for improving the estimation of correlations have to date included the shrinkage approach, Corpcor [1]. However, this method is applied uniformly to the full correlation matrix. In practice, recent graphical model approaches have used the block diagonal form of the covariance matrix to improve computational efficiency and interpretation [2]. In our case, we assume that sets of genes, within an operon for prokaryotes or under the control of one transcription factor for eukaryotes, will strongly correlate with each other. Such correlated sets are then represented by a single block, and the full correlation matrix is then split into separate blocks or transcriptional units. Consequently, a block diagonal structure of the covariance matrix is used to model this. A different covariance matrix in block diagonal form is used for each condition present in the data, meaning that different transcriptional units may be present in a case compared to a control matrix.

In conjunction with assuming the block diagonal form of the covariance matrix, we have taken an empirical Bayes approach to model the covariance, and similarly calculate correlations. This approach uses the data to generate the prior information matrix and borrows information from across the genes to estimate covariances in such a way as to lower the false discovery rates (FDRs). This is important in downstream analysis where we would like to limit the number of false discoveries when identifying candidates to test experimentally.

## Implementation

We use an empirical Bayes approach to infer covariances and by simple extension, correlations. The theoretical basis of this, using conjugate priors, is derived in [3]. In that paper, Champion et al. use an independence prior, and a flat prior with constant covariance. We introduce a new prior that is a mixture of these two extremes, the block diagonal prior.

Bayes theorem relates the posterior distribution of the parameters given the data *p*(*Θ*|*X*) to the likelihood of the data *p*(*X*|*Θ*) and the prior distribution of the parameters *p*(*Θ*) via

$$ p(\Theta|X) \propto p(X|\Theta)p(\Theta) $$

We assume the data *X* are multivariate normal with sample size *n*, covariance matrix *Σ*, mean *μ* and number of variables (genes) *p*, and thus the likelihood is proportional to

$$p(X|\Theta) \propto |\Sigma|^{-\frac{1}{2}}{\exp}\left\{-\frac{1}{2}(x-\mu)^{T}\Sigma^{-1}(x-\mu)\right\} $$

We wish to obtain a Bayesian estimate for the covariance matrix *Σ*, which we denote by *η*, and we assume that *μ* is known. The conjugate prior for estimating a covariance matrix (*η*) is an inverse Wishart distribution. We adopt the same parameterisation as [3]: *η*∼*W* ^−1^(*ψ*,*λ*) where *ψ* is the mean of the Inverse Wishart distribution ($\frac {\lambda z}{\lambda }$) and *λ*+*p*+1 are the degrees of freedom. The probability density function (pdf) for the inverse Wishart is proportional to

$$ p(\Theta) \propto |\eta|^{-(\lambda +2p+2)/2)}{\exp}\left\{-\frac{1}{2}\text{tr}(\lambda z \eta^{-1})\right\} $$

To obtain the joint posterior distribution we multiply the likelihood for sample data (which is multivariate normal) with the prior. Let $S=\sum {(x'-\mu ')(x'-\mu ')^{T}}$, where *x* ^′^ is the sample data and *μ* ^′^ the sample mean. Then the joint posterior distribution *p*(*Θ*|*X*) is proportional to

$$|\eta|^{-(n+\lambda+2p+2)/2}\exp\left\{-\frac{1}{2}\text{tr}(\eta^{-1}(\lambda z + S))\right\} $$

which is an inverse Wishart distribution with parameters $\left (\frac {\lambda z+S} {\lambda +n},\lambda +n\right)$. We then estimate *η* by *η* ^0^, the expected value of the distribution:

$$\begin{array}{*{20}l} \hspace{70pt} \eta^{0} = \frac{\lambda z + S}{\lambda +n } \end{array} $$

From this we can see that the inverse Wishart requires two hyperparameters, the matrix z and scalar *λ*.

### Calculating hyperparameters

In empirical Bayes methods both hyperparameters *λ* and *z* are estimated from the data, rather than using a hierarchical model and assigning a prior distribution to each of the parameters, or by having to choose the parameter values where little prior knowledge is available. In choosing the matrix *z* we are looking for an appropriate prior matrix for the covariance matrix *η*. The Inverse Wishart distribution requires that the matrix *z* is positive definite, block diagonal matrices are positive definite if and only if each of the blocks are positive definite. Therefore we construct a block diagonal prior matrix where each of the blocks have a constant (non perfect) correlation. The estimated correlation matrix from this method *η* ^0^ is then a mixture of the sample correlation matrix S and the block diagonal prior matrix z.

As we are using a block diagonal matrix for *z* we have added an extra level of estimation to the model over other methods that fix the structure of the prior matrix *z* a priori as, for example, the independence or flat correlation matrix. By using the block diagonal matrix the different groupings or blocks need to be determined for each data set separately. There are three methods for generating the blocks for the matrix z available in the package covEB. The first is to provide a list of the block assignments for each variable in the covariance matrix. Each entry in the list should comprise a set of variables that together will form one block. The algorithm then uses the average correlation within these blocks to calculate a constant value for this block in the matrix z, with all elements outside the block set to zero.

The second method is to provide a threshold parameter (or correlation level). This could be based on prior knowledge or calculated using an existing method for thresholding covariance matrices such as those described in Bickel et al. [4]. Given this correlation level (used for simplicity to allow the user to set thresholds in [-1,1]), we set all elements of the sample correlation matrix below this threshold to zero and identify the block diagonal structure. This is done by treating the correlation matrix as a weighted graph and using the cluster function in the R package igraph [5] to separate the graph into disjoint subnetworks such that each element in one subnetwork is completely separate from all elements in the other subnetworks, that is the correlation between them is zero. Each subnetwork then represents one block in the block diagonal matrix.

The third method requires no prior information from the user, instead the Akaike’s Information Criteria (AIC) metric is used to select the threshold level that determines the block diagonal structure. The AIC is defined as *AIC*=2*p*−2 ln(*L*), where *p* are the number of parameters estimated in the model and ln(*L*) is the log-likelihood of the data given the model. In our case, we calculate the likelihood of observing the given covariance matrix for different block diagonal matrices (models). We assume the data is from a multivariate normal distribution with covariance matrix *Ω*. *Ω* is the diagonal matrix calculated (as outlined above) for different threshold values, this threshold value is chosen to minimise the AIC statistic.

Once the groupings are known, it remains to find the correlation level within each block that together with the groupings will define the prior matrix *z*.This is done by averaging the correlations within each block to give a constant correlation value for that block in the prior matrix z. For example, in the block diagonal matrix z below we have 5 blocks, membership of variables in these 5 blocks having being determined by one of the three methods outlined above.

$$\begin{array}{*{20}l} \hspace{60pt} \left(\begin{array}{ccccc} \delta_{1} & 0 & 0 & 0 & 0 \\ 0 & \delta_{2} & 0 & 0 & 0 \\ 0 & 0 & \delta_{3} & 0 & 0 \\ 0 & 0 & 0 & \delta_{4} & 0 \\ 0 & 0 & 0 & 0 & \delta_{5} \end{array} \right) \end{array} $$

If, for example block 1 contains 4 variables (or genes) then, *δ*_1_ (the first block in the matrix z) takes the form:

$$\hspace{70pt} \left(\begin{array}{cccc} 1 & \gamma & \gamma & \gamma \\ \gamma &1 & \gamma & \gamma \\ \gamma & \gamma & 1 & \gamma \\ \gamma & \gamma & \gamma & 1 \end{array} \right) $$

and *γ* is estimated from the data as the average of the sample correlations between the four variables.

Given the current estimate of *z* we then calculate *λ* using the following approximation suggested by [3]:

$$E((\rho_{ij}[\!\eta]-\rho_{ij}[\!z])^{2}) \simeq \frac{(1-\rho_{ij}[\!z]^{2})^{2}}{\lambda+3} \text{for} i \neq j, $$

where *ρ*_*ij*_[ *η*] is the correlation based on *η*. We approximate *ρ*_*ij*_[ *η*] by the sample correlations, and *ρ*_*ij*_[ *z*] are the correlations based on *z* for the selected value of *γ*. See [3] for full details of this derivation. However, briefly, this result follows from the distribution of the sample correlations *ρ*_*ij*_[ *η*] that is a two parameter distribution whose mean is *ρ*_*ij*_[ *z*]. Given the distribution of the *ρ*_*ij*_[ *η*], the variance *var*(*ρ*_*ij*_[ *η*])=*E*((*ρ*_*ij*_[*η*]−*ρ*_*ij*_[ *z*])^2^) is approximated using the moments of this distribution.

### Simulated data

For the simulated data we use the method of Hardin to generate block diagonal matrices [6]. We generated 100 sample covariance matrices for 50 genes, each covariance matrix had a block diagonal structure with 5 blocks of different sizes. Correlations within blocks were set to 0.7, random samples were generated for each of these correlation matrices for three different sample sizes of 10, 15 and 20. We include a simulated dataset under these parameters in the R package covEB. We denote the empirical Bayes method using a block diagonal prior as EB, for comparison two other methods are used to calculate the correlation matrix, the Pearson correlation matrix, and the Corpcor method of Schäfer et al. are used to estimate elements within blocks and all the elements outside these blocks are set to zero. We calculated the Frobenius norm between each of the estimation methods and the true block diagonal matrix. We estimated the correlation matrices and calculated the average false and true positive rates and their standard deviations over the 100 simulations, for each of the methods mentioned above.

### Biological data

The *Bacillus subtilis* data were taken from the paper [7]. A subset of 19 samples that are clustered according to the affinity propagation clustering with Euclidean distance was used. The genes were filtered leaving those with variance above the median. For each method we used known transcriptional unit information from BSubCyc (http://www.bsubcyc.org) to generate the prior groupings, selecting 56 known transcriptional units each with at least 5 genes in them.

## Results and discussion

First we compared the estimation of elements within blocks only for the three methods using the Frobenius norm, assuming known groups. The estimation using the EB method resulted in a matrix that was closer to the true matrix within blocks with similar standard deviation across simulations when compared to all of the other methods. This is shown in Table 1 which gives the average and standard deviation of the Frobenius norm between the estimated and true matrix over 100 simulations, for example when n=15, the EB method has a distance (according to the Frobenius norm) of 2.81, compared to 4.10 for the Pearson matrix and 17.99 for the Corpcor method. When the groupings are known, using the Pearson estimation within blocks and setting elements outside blocks to zero will give a smaller distance than the EB method. This is because the Frobenius norm of the elements outside blocks will be exactly zero by construction, in contrast, the EB method will shrink these elements to zero rather than setting them to be exactly to zero, and gives and increase to 7.59 from 2.81 when *n*=15. Therefore, in the case where the groupings are known, we would expect using the EB method within blocks and setting elements outside blocks to zero will give the closest result to the true matrix under the Frobenius norm.

**Table 1:** Simulation results for the empirical Bayes method with known groupings (blocks)

	Pearson block	Corpcor block	EB block	EB
Mean *n*=10	5.67	20.41	4.08	9.23
Sd *n*=10	1.49	1.44	1.64	1.72
Mean *n*=15	4.10	17.99	2.81	7.59
Sd *n*=15	0.98	0.93	1.05	1.13
Mean *n*=20	4.08	18.42	2.69	7.39
Sd *n*=20	0.76	0.80	0.75	0.90

We calculate the Frobenius norm between the estimated and true matrix for elements within the blocks. The estimates for the full matrices are the same for the Pearson and the Corpcor methods by construction, in contrast there is a small increase in the Frobenius norm for the EB method. This is due to the fact that for the Pearson and Corpcor method we set all elements outside the known blocks to zero whilst the EB method sets these elements to zero in the prior matrix, rather than the final estimated matrix

As a second test we also generated group assignments using the thresholding method (where the threshold level is known) and compared the EB estimate and the matrices estimated using either Pearson or Corpcor within blocks with elements outside blocks set to zero. The results given in Table 2 showed improvement of the EB method over the Corpcor and Pearson estimation methods. In particular, the EB method shows the largest improvement over the other methods when the sample size is smallest (*n*=10). For comparison purposes we also show the results from the EB method using no prior information (EB AIC). For this estimate the threshold level is selected from the data using the AIC metric. For *n*=10 the EB AIC method is still closer to the true matrix than any of the other methods, and has comparable distances for the larger sample sizes.

**Table 2:** Simulation results with known threshold level and groups then estimated from the data

	Pearson	Corpcor	EB	EB AIC
Mean *n*=10	19.27	23.48	17.65	17.86
Sd *n*=10	3.42	3.70	3.34	3.14
Mean *n*=15	12.30	17.45	12.72	13.89
Sd *n*=15	4.34	2.04	3.81	3.21
Mean *n*=20	10.52	17.52	11.46	13.19
Sd *n*=20	3.96	1.81	3.28	2.81

We calculate the Frobenius norm between the estimated and true matrix for the full matrix. This shows a particular improvement when sample size is small (*n*=10) for the EB method, as the matrix is closer to the true matrix than for either the Pearson or Corpcor method. We also compare the result when the AIC method is used to estimate the threshold level (EB AIC). This method shows similar improvements when *n*=10 and comparable results to the other methods with the larger sample sizes. Indicating the AIC function has provided a reasonable estimate of the threshold level

With simulated data, we also compared the FDR and TPR at a 5% significance level for each of the three methods. The first three columns of Table 3 compare the results when the groups are known. In this case there are zero false positives, which occur by definition of the covariance matrix for the Corpcor and Pearson estimates, but are also matched by the EB method. When the groups are not known, but the threshold value is, the next three columns show improved or similar FDR for the EB threshold method, particularly at the smaller sample sizes. However, there is a trade-off as the TPR is lower for EB block when *n*=10, though it is comparable for *n*=15 and improved for *n*=20. We also compare the rates where no prior information was used and the AIC method was used instead to select the threshold (EB AIC). This is shown in the final column, although we expect there may be further errors due to the extra level of estimation, this method again shows close results to the EB threshold method, meaning that at small sample sizes it has improved FDR over other methods but with lower TPR rates that improve as the sample size increases.

**Table 3:** Simulation results with known groups (first three columns) and using a known threshold level to estimate groups or blocks from the data (threshold) and the EB AIC method for when neither the threshold or groups are known

		EB	Corpcor	Pearson	EB threshold	CorpCor threshold	Pearson threshold	EB AIC
*n*=10	FDR mean	0.00	0.00	0.00	0.04	0.26	0.16	0.04
	FDR sd	0.00	0.00	0.00	0.09	0.12	0.09	0.09
	TPR mean	0.70	0.54	0.66	0.39	0.56	0.66	0.36
	TPR sd	0.21	0.12	0.15	0.21	0.13	0.15	0.21
*n*=15	FDR mean	0.00	0.00	0.00	0.09	0.16	0.13	0.06
	FDR sd	0.00	0.00	0.00	0.11	0.12	0.11	0.11
	TPR mean	0.90	0.32	0.83	0.75	0.59	0.81	0.61
	TPR sd	0.10	0.08	0.10	0.19	0.19	0.12	0.21
*n*=20	FDR mean	0.00	0.00	0.00	0.11	0.11	0.10	0.10
	FDR sd	0.00	0.00	0.00	0.10	0.10	0.09	0.10
	TPR mean	0.97	0.44	0.90	0.91	0.55	0.88	0.79
	TPR sd	0.04	0.09	0.06	0.09	0.14	0.08	0.11

We calculate the FDR and TPR for all the variables by comparing them to the true matrix, significance of correlations was determined using a t-test. We see improved or comparable FDR’s for the EB methods across all sample sizes. There is a particular improvement for *n*=10, however, there is a trade-off in terms of lower TPR. However, together we would still expect the EB method to find high value interactions as significant, which is important in designing downstream validation experiments

For the *Bacillus subtilis* data, we calculated the FDR rates based on the known transcriptional units for the EB method at 6%, this is an acceptable error level for most experiments that usually aim for a FDR of 5%. By construction with the Pearson and Corpcor method we set elements outside blocks to zero, therefore comparing these to the prior information the FDR for both methods is zero. As a comparison, without imposing the block diagonal structure on these matrices, the Pearson and Corpcor method had an FDR of 20% and 39% respectively. The EB method also resulted in similar or improved true positive rates of 93% compared to 92% and 43% for Pearson and Corpcor respectively.

**Fig. 1:**
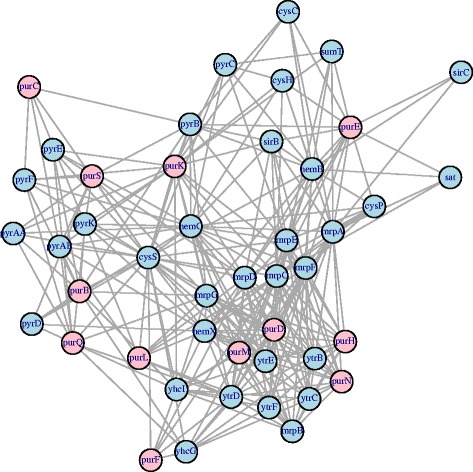
This is an example of the EB estimated network containing a transcriptional unit regulated by PurR (pur(E,K,B,C,S,Q,L,F,M,N,H,D)), that are shown in *pink*. As the EB method was the only method used that allowed for connections between transcriptional units, we can see the additional information gained from this by those elements connected to this transcriptional unit. These include the pyr transcriptional unit that is also known to be regulated by PurR

One potential downside of setting elements outside known transcriptional units to zero (as we did with the Pearson and Corpcor estimates) is that the inference may miss interactions between transcriptional units supported by the data. By using the t-test on the significance of the correlations from the EB estimate, we identified edges between genes in different transcriptional units at a 5% significance level. As an example we looked at the transcriptional unit of Pur(E,K,B,C,S,Q,L,F,M,N,H,D) that is regulated by PurR, this is shown in Fig. 1 and its genes are coloured pink. This transcriptional unit also has significant connections to the transcriptional unit of pyr(AA,AB,B,C,D,E,F,K) that is also known to be regulated by PurR. Other connected genes in the same transcriptional units include ytr(B,C,D,E,F), regulated by Sigma Factor A and the transcriptional regulator YtrA of the GntR family. The transcriptional unit combining cys(C,H,P), sat, sumT and sir(B,C), also regulated by Sigma Factor A. Hem(B,C,X) that is regulated by PerR and the transcriptional unit containing mrp(A,B,C,D,E,F,G) and two genes yhcG and yhcI in the same operon, neither of these units currently have any known regulators.

## Conclusions

Above we show that the EB method is closer to the blocks within the true matrix, as calculated using the Frobenius norm, for each of the sample sizes when using either the known groupings or those generated from the simulated data (using a known thresholding level). Further using a 5% significance level the EB method has lower false discovery rates than the Pearson covariance matrix and existing Corpcor method.

The simulation results indicate that we are able to improve the false discovery rates when estimating correlations that can be used in downstream analysis. The EB shows particular improvements when the sample size was as small as ten replicates. This is important as many experiments have comparable levels of replication by necessity.

Further, controlling the false discovery rate is particularly useful when the network inferences are used to drive experimental hypotheses, as we are interested in identifying the highest possible value links for subsequent laboratory analysis. The EB method was also able to find connections between transcriptional units sharing the same regulator (PurR). This shows how the EB method is flexible, controlling error rates whilst also allowing significance connections between genes or transcriptional units that are supported by the data.

## Availability of data and materials

**Project name**: covEB

**Project home page**: http://bioconductor.org/packages/covEB/

**Operating system(s)**: Platform independent

**Programming language**: R

**Other requirements**: R (≥ 3.3)

**License**: GPL-3

**Any restrictions to use by non-academics**: None

## Abbreviations

cys(C,H,P): Cysteine -C,H,P
EB: Empirical Bayes
FDRs: False discovery rates
hem(B,C,X): Heme -B,C,X’
PerR: Transcriptional repressor of the peroxide region
PurR: Purine biosynthesis
TPR: True positive rate
YtrA: Transcriptional regulator YtrA (GntR family)
